# A Simple, Reproducible and Low-cost Simulator for Teaching Surgical Techniques to Repair Obstetric Anal Sphincter Injuries

**DOI:** 10.1055/s-0038-1668527

**Published:** 2018-08

**Authors:** Roxana Knobel, Lia Karina Volpato, Liliam Cristini Gervasi, Raquel de Almeida Viergutz, Alberto Trapani

**Affiliations:** 1 Department of Ginecology and Obstetrics, Universidade Federal de Santa Catarina, Florianópolis, SC, Brazil

**Keywords:** natural childbirth, suture techniques, anal sphincter/injuries, simulation training, parto natural, técnicas de sutura, esfíncter anal/lesões, treinamento de simulação

## Abstract

**Objective** To describe and evaluate the use of a simple, low-cost, and reproducible simulator for teaching the repair of obstetric anal sphincter injuries (OASIS).

**Methods** Twenty resident doctors in obstetrics and gynecology and four obstetricians participated in the simulation. A fourth-degree tear model was created using low-cost materials (condom simulating the rectal mucosa, cotton tissue simulating the internal anal sphincter, and bovine meat simulating the external anal sphincter). The simulator was initially assembled with the aid of anatomical photos to study the anatomy and meaning of each component of the model. The laceration was created and repaired, using end-to-end or overlapping application techniques.

**Results** The model cost less than R$ 10.00 and was assembled without difficulty, which improved the knowledge of the participants of anatomy and physiology. The sutures of the layers (rectal mucosa, internal sphincter, and external sphincter) were performed in keeping with the surgical technique. All participants were satisfied with the simulation and felt it improved their knowledge and skills. Between 3 and 6 months after the training, 7 participants witnessed severe lacerations in their practice and reported that the simulation was useful for surgical correction.

**Conclusion** The use of a simulator for repair training in OASIS is affordable (low-cost and easy to perform). The simulation seems to improve the knowledge and surgical skills necessary to repair severe lacerations. Further systematized studies should be performed for evaluation.

## Abstract (pt)

**Resumo:**

**Objetivo** Descrever e avaliar a utilização de um simulador simples, de baixo custo e reprodutível para o ensino de sutura de lacerações perineais de 4° grau.

**Métodos** Participaram da simulação 20 residentes de ginecologia e obstetrícia e quatro profissionais especialistas. Um modelo de laceração de 4° grau foi criado com materiais de baixo custo (preservativo simulando a mucosa retal, tecido de algodão simulando o esfíncter anal interno e carne bovina simulando o esfíncter anal externo). O simulador foi inicialmente montado com ajuda de fotos anatômicas, para estudar a anatomia e o significado de cada componente do modelo. A laceração foi criada e suturada, utilizando técnicas de borda a borda e de sobreposição do esfíncter anal.

**Resultados** O modelo custou menos de R$ 10,00 e foi montado sem dificuldade, aprimorando os conhecimentos dos participantes sobre anatomia e fisiologia. As suturas das camadas (mucosa retal, esfíncter interno e esfíncter externo) foram realizadas seguindo a técnica cirúrgica. Todos os participantes ficaram satisfeitos com a simulação e consideraram que esta melhorou seus conhecimentos e habilidades. Entre 3 a 6 meses após o treinamento, 7 participantes presenciaram em sua prática lacerações graves e relataram que a simulação foi útil para a correção cirúrgica.

**Conclusão** A utilização de um simulador para treinamento de sutura de lacerações obstétricas graves é acessível (baixo custo e fácil execução). A simulação parece aprimorar conhecimentos e habilidades cirúrgicas para sutura de lacerações graves. Mais estudos sistematizados devem ser realizados para avaliação.

## Introduction

Severe perineal laceration involving the anal sphincter is an important complication of vaginal delivery. Its incidence is used as a safety marker in childbirth, and it can be used to evaluate an institution or region.^1^ ^2^ ^3^ The reported incidence varies according to hospital, country, obstetric practice, and diagnosis, ranging from 1.2 to 6% of births.^3^ ^4^ ^5^

An obstetrician should be able to diagnosis and adequately correct obstetric anal sphincter injuries (OASIS).^6^ ^7^ However, there are few training opportunities for resident doctors to practice surgical skills in vivo, and there is a lack of knowledge regarding the recognition and repair of OASIS.^8^ Considering that the procedure has a learning curve,^9^ and lacerations corrected by experienced obstetricians or specialized surgeons are more likely to have a proper result,^7^ the importance of training in this repair stands out.

The use of simulators and simulation environments for teaching health professionals is well established.^9^ ^10^ ^11^ ^12^ They replicate a clinical scenario, with a controlled situation, allowing a detailed observation of the students in action, with feedback and the possibility of several repetitions without any harm to patients.^9^ ^10^

Overall, the quality of the evidence about simulation-based learning (SBL) is low, but it suggests that the method is effective and leads to better and longer-lasting results compared with traditional teaching.^11^ ^13^ In surgical training, for instance, it may reduce costs and improve clinical outcomes.^9^ ^14^

While simulated environments and high-fidelity simulators have proved to be useful, there are barriers to their use in teaching, mainly concerning their cost.^9^ ^10^ ^15^ There is no evidence that a hyper-reality simulator improves the learning of participants.^16^ Therefore, low-cost simulators can be effective in the teaching and learning process,^9^ ^14^ with characteristics demonstrated even in obstetric situations.^9^ ^15^ ^17^ Several simulators aimed to improve surgical skills in the repair of vaginal lacerations and OASIS were described, all with positive results.^9^ ^18^ ^19^ ^20^ ^21^ ^22^

The objective of the present study was to describe and evaluate a simple, low-cost and reproducible simulator, adapted to the Brazilian reality, for teaching OASIS repair.

## Methods

This is an observational qualitative-quantitative research. The research is part of the project “handmade simulators for teaching in obstetrics”, which was developed by the authors and seeks to create, discover, compile, and disseminate the possibilities of using simulators and accessible simulations (http://saudesimuladores.paginas.ufsc.br/).

The simulations took place in classrooms of two public hospitals with medical residents, both located in the southern region of Brazil. They lasted approximately 2 hours each and were done through classes and clinical discussions with residents during the year of 2017. Participants included gynecology and obstetrics residents and experts in the area. There were ∼ 12 participants per simulation, and some respondents did the simulation twice. The criteria for participating in the study were: being a gynecologist and obstetrician resident or expert, participating in the simulation and agreeing to complete the questionnaire, and signing the informed consent form.

**Fig. 1:**
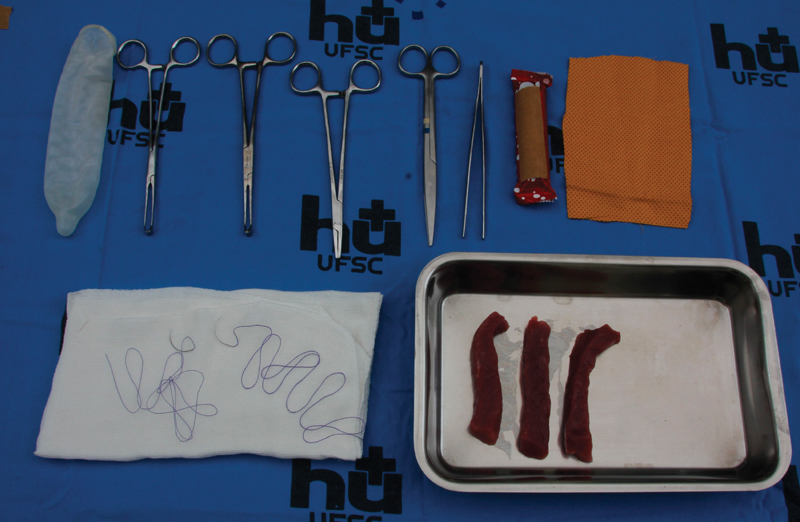
Material used for simulator assembly and simulated laceration repair - Chocolate bar, condom, 15 cm × 10 cm cotton cloth flap, beef strips ∼ 1 cm × 1 cm × 8 cm, surgical material (tweezers, needle holder, scissors, Allis clamp) and suture.

The simulation model was created based on existing models.^9^ ^22^ To assemble the simulator, anatomical photos were used to determine the anatomical structures and the function of each component of the model. The material needed for the assembly included: chocolate bar or similar; condom (preferably without lubricant); 15 cm × 10 cm cotton cloth flap; beef strips of ∼ 1 cm × 1 cm × 8 cm; surgical material (tweezers, needle holder, scissors, Allis clamp) and suture (Fig. 1). The beef was fat-free and had the longest fibers running longitudinally to simulate the sphincter fibers.

**Fig. 2:**
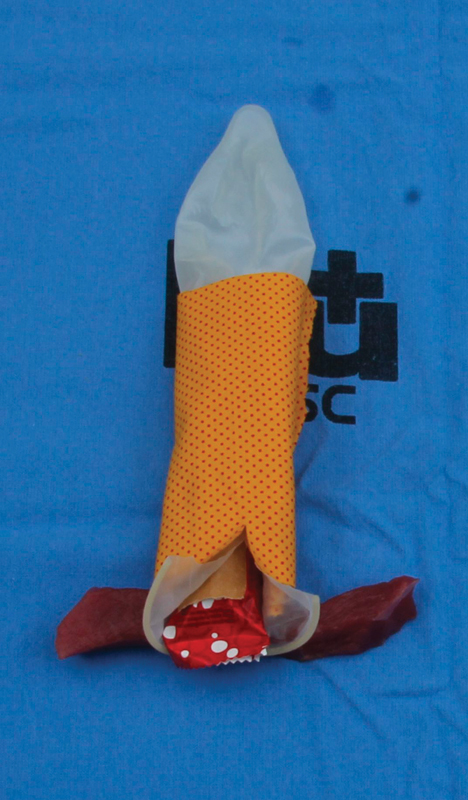
Representation of the severe perineal laceration in the simulator.

A condom with a chocolate bar inserted in it represented the rectal mucosa and the intestinal contents (necessary to give volume to the model). The internal anal sphincter is a bright, fibrous structure that, when completely torn, generally retracts laterally. Suturing this structure separately from the external anal sphincter improves the posterior results,^5^ ^23^ so it was decided to include it in the simulation, represented by a flap of cotton cloth. The beef strip represented the external anal sphincter. After assembling the model, a laceration was created (Fig. 2).

**Fig. 3:**
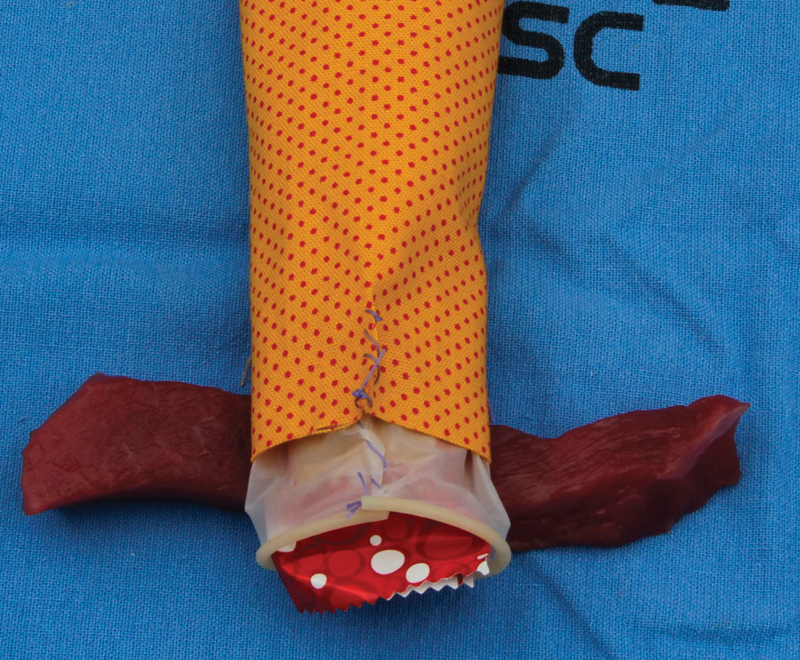
Representation of the rectal mucosa and the internal anal sphincter sutured with a simple continuous suture.

**Fig. 4:**
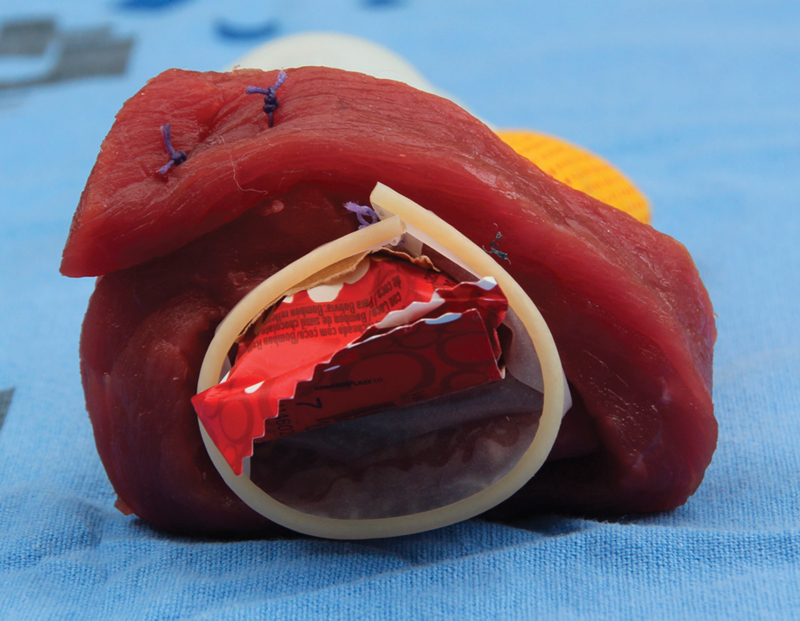
Representation of the external anal sphincter repair: overlapping plication.

The practical aspects of diagnosing and suturing severe lacerations include the need to evaluate the sphincter and the rectal mucosa after the delivery, adequate anesthesia, positioning of the patient, illumination, a good surgical field, and antisepsis.^23^ ^24^ ^25^ The most appropriate wires for each anatomical layer were presented. The torn anal mucosa is repaired using a continuous (nonlocking) 3-0 or 4-0 braided polyglactin on a tapered needle; a monofilament suture such as poliglecaprone 25 is also acceptable. The internal anal sphincter should be properly identified and repaired as a separate layer (Fig. 3) using a continuous 3-0 polyglactin suture or a 3-0 monofilament synthetic suture (for example, poliglecaprone 25) on a tapered needle.^24^ ^25^ The external anal sphincter was sutured with end-to-end techniques or overlapping plication (Fig. 4) using interrupted or figure-of-eight sutures; 2-0 or 3-0 polydioxanone or 2-0 polyglactin suture on a tapered needle.^24^ ^25^ In the simulation, to reduce costs, yarns that were past due or cheaper, such as catgut, were used.

All the participants answered a questionnaire three to six months after the simulation. The questionnaire sought to evaluate the experience, satisfaction, and learning with the simulator and to determine if the participants had encountered any cases of severe perineal laceration after the simulation and whether they had noticed changes in their surgical performance.

The quantitative variables were analyzed with descriptive statistics, and the qualitative variables were categorized according to their content. The local ethics committee approved the research project.

## Results

The simulator was created at a cost of approximately R$10.00. Twenty resident doctors and four expert obstetricians participated in the simulations. Only one resident who participated did not respond to the questionnaire. The mean age of the participants was 30.83 years old (standard deviation [SD] = 6.99), and the time since graduation in medicine was 4.5 years (SD = 5.64). Among the participants, five were in the first year of residence; seven were in the second; and eight were in the third. The time of experience of the experts varied between 10 and 20 years at the time of the simulation.

All the participants were satisfied with the training and considered that the simulation improved their knowledge and skills for correction of severe perineal lacerations. In the open questions, greater security and confidence in the case of necessity to perform the suture were the most cited categories.

The majority (78%) of the participants considered that the simulator was effective in replicating the anatomical structures, with inherent limitations to the model.

- “The thickness of the layers is very reliable and simulates the technical difficulties of the actual tear.” (Expert 3)
- “It allows visualizing the anatomy, mainly the texture/thickness of the external anal sphincter.” (Resident 14)
- “I have done training with 100% synthetic material, and this is closer to reality.” (Resident 8)
- “It is very difficult to simulate the anatomy; the model is very simplified.” (Resident 4)
- The majority (69%) of the participants also considered that they were not immersed in the experience (as if it were a real service).
- “The class was relaxed; we played, made mistakes, and we did it again. In practice, nervousness and responsibility weigh heavily on the procedure.” (Resident 18)
- “Remember step-by-step in case of necessity, but far from being a real situation.” (Resident 3).

Table 1 shows the self-evaluation of the participants regarding their preparedness to repair OASIS before and after the simulation.

**Table 1:** Self-evaluation of the participants of the simulation regarding their preparedness to suture a severe laceration

Do you feel ready to repair OASIS?	Before simulation	After simulation
	n (%)	n (%)
No	14 (60.87)	1 (4.35)
Partially	5 (21.74)	7 (30.43)
Yes	4 (17.39)	15 (65.22)

*p* < 0.05.

Abbreviation: OASIS, obstetric anal sphincter injuries.

Four resident doctors attended cases of severe perineal rupture after participating in the simulation and considered that the training helped them remain calm and know how to proceed, in addition to having improved their surgical skills. Of the four experts, three attended serious lacerations after participating in the simulation and also considered that they were more confident and calmer when performing the procedure.

- “I felt more confident; I was able to better identify the structures involved.” (Resident 5)
- “... the suture becomes more automatic.” (Expert 1).

## Discussion

There are several models for training the repair of severe perineal lacerations, the most described being synthetic,^8^ ^26^ or pigs' or goats' anal sphincter.^8^ ^18^ In addition, some authors describe the use of a set with cattle' or pork' tongue with other meats or coupled with synthetic material.^19^ ^21^ The simulation of the internal anal sphincter is not performed in most models of this type.^9^ ^22^ In the model used in the present study, the anal sphincter was simulated using a flap of cotton cloth.

There is no need for the simulation to be ultra-realistic; a more simplified scenario can achieve the same objectives and is more accessible and reproducible at low cost.^10^ ^21^ In the present case, the model is simple, affordable, and achieves the objectives (to improve the knowledge and skills for suturing severe perineal laceration). However, it was not possible to accurately reproduce the anatomy, which other models do more effectively (such as the use of goat or pig anal sphincter).^8^ ^18^ Regardless, no model reproduces the human anatomy perfectly.^18^

The format of the simulation (limited time in a classroom, several people training at the same time) did not allow an immersion in the experience; the students did not feel the simulation as real. Although the immersion in the simulation is important in some contexts (emergencies, teamwork),^9^ ^10^ other studies on suture of severe lacerations do not mention immersion as a variable, probably because the focus is a specific surgical skill. Other formats that simulate the surgical environment or have separate stations can help to improve the experience in this regard.

The improvement in surgical skills was achieved and assessed only by the self-evaluation of the participants, a method also used in other researches.^19^ ^21^ In other studies, there was an improvement of skills, and the evaluation was done objectively with tests and/or objective structured assessment of technical skills (OSATS).^8^ ^19^ ^21^ ^26^

A simple, accessible, and easily reproducible simulator for suture training for severe perineal laceration repair was created and used. All the participants enjoyed the simulation and assessed that their knowledge and skills improved. At least seven of the participants had to attend serious lacerations after participating in the simulation and reported feeling more confident and secure. Improved self-confidence to care for a case is described in other studies.^8^ ^19^ ^21^ ^26^ It is believed that, because of the simplicity of the simulator, it can be widely replicated. The training can be done by more obstetricians and resident doctors, improving the results of corrections of severe perineal lacerations.

The simulation was done in class time, with no need for a specific environment, which on the one hand is a disadvantage, since it did not allow students to immerse in the simulation. On the other hand, it can be seen as an advantage, since it can be done in all institutions, without the need of more a complex preparation.

The present study has some limitations. Only the apprentices themselves evaluated the knowledge and skills acquired in a single moment. The teachers who guided the simulation belong to the institution and are known to the resident doctors. Although the questionnaires are anonymous, there may be a courtesy bias in the answers. For future investigations, a pre- and postsimulation evaluation is suggested, either with a theoretical test or with an OSATS and evaluation sometime later, to evaluate the retention of knowledge. It was possible, however, to notice changes in the behavior of the learners (level 3 on the Kirkpartick scale, defined as behavioral changes in the work environment attributed to the learning opportunity).^27^

## Conclusion

The use of a simulator for OASIS repair is affordable (low-cost and easy to perform) and can be an alternative for resident doctors and expert training. The simulation seems to improve the knowledge and surgical skills to suture severe lacerations. Further systematized studies should be performed for evaluation.
